# Reaching a Tipping Point: A Qualitative Exploration of Quality of Life and Treatment Decision-Making in People Living With Benign Prostatic Hyperplasia

**DOI:** 10.1177/10497323221129262

**Published:** 2022-09-26

**Authors:** Margaret Husted, Debra Gray, Sarah E. Golding, Richard Hindley

**Affiliations:** 1 Department of Psychology, 405729 University of Winchester, Winchester, UK; 2 Urology Department, 9946 Hampshire Hospitals NHS Foundation Trust, Winchester, UK

**Keywords:** benign prostatic hyperplasia, prostate enlargement, treatment decisions, quality of life, lower urinary tract symptoms, erectile dysfunction

## Abstract

Benign prostatic hyperplasia (BPH) is a common condition amongst older men and is associated with lower urinary tract symptoms and erectile dysfunction; these symptoms can be burdensome and negatively affect quality of life. Various surgical and pharmaceutical treatment options exist but there is a paucity of qualitative research exploring men’s decision-making when seeking BPH treatment. This study qualitatively explored men’s experience of living with BPH and seeking treatment for BPH. Twenty men (aged 52–75) were recruited from outpatient urology clinics at a hospital trust in Southern England. Data were collected using semi-structured interviews (via video or telephone call) and were audio-recorded; transcripts were analysed using thematic analysis. Four themes were generated: ‘Impacts are about more than just physical symptoms’, ‘The path towards treatment’, ‘The process of information gathering’ and ‘Navigating hopes, fears and uncertainty’. Results indicate most men appear to seek treatment for BPH following a gradual, and sometimes lengthy, period of deterioration in symptoms; for some men, however, treatment seeking follows an acute episode of sudden or severe symptoms. The decision to proceed with surgical or minimally invasive treatment options appears to be dependent on men reaching a tipping point; they no longer perceive their symptoms as tolerable and feel their ability to cope with symptoms is reduced. Men each bring their own set of concerns and preferences about side effects and risk-benefit profiles of different treatments. Clinicians need to be sensitive to these individual differences and incorporate them into shared decision-making for choosing between treatment options for BPH.

## Introduction

Benign prostatic hyperplasia (BPH) is a common condition affecting approximately 50%–80% of men over the age of 50 (Berry et al., 1984), with prevalence increasing as men age (Joseph et al., 2003; Kupelian et al., 2006; Martin et al., 2011; Rosen et al., 2003). Benign prostatic hyperplasia is associated with significant troublesome symptoms, such as lower urinary tract symptoms (LUTS, e.g. nocturia, increased frequency and urgency of urination and retention) and erectile dysfunction (ED) (Girman et al., 1999; Kupelian et al., 2006; Malde et al., 2019), which can have psychological, emotional and sexual impacts that negatively affect men’s quality of life (QoL) (Andersson et al., 2004; Girman et al., 1999; Kupelian et al., 2006; Welch et al., 2002). Bothersome symptoms are associated with reduced mental health and psychological wellbeing (Boyle et al., 2003; Ikenwilo et al., 2018; Obayashi et al., 2017; Pinto et al., 2015). In one USA survey, severe LUTS had a greater impact on QoL than did living with gout, hypertension, angina or diabetes (Welch et al., 2002).

### Treatment Decision-Making in Men with BPH

Dependent upon the severity of symptoms, the size of the prostate and other comorbidities, treatment options for BPH include watchful waiting, lifestyle advice, medication, minimally invasive surgical techniques (MISTs) and surgeries (Oelke et al., 2013). The current ‘gold standard’ surgical treatment in the UK is transurethral resection of the prostate (TURP). Transurethral resection of the prostate represents about 80% of the 25,000 BPH operations performed each year (Worthington et al., 2017), although other less invasive surgical options are increasingly available (Gacci et al., 2011; Oelke et al., 2013; Rieken & Kaplan, 2018).

Although treatment decision-making in men with prostate cancer has been widely studied (Løwe et al., 2021; Showalter et al., 2015; Volk et al., 2014; Zeliadt et al., 2006), there is a paucity of, particularly qualitative, research exploring factors that influence treatment decision-making amongst men with BPH (De Nunzio et al., 2017). Research that does exist indicates many men want to avoid surgery and may be prepared to wait longer for their symptoms to improve from medication, if this reduces their risk of surgery (Emberton et al., 2008; Ertel et al., 2016; Malde et al., 2021; Watson et al., 2004). However, alternative research found men were more concerned about potential catheterisation for acute urinary retention, than they were about surgery for BPH (Kawakami & Nickel, 1999). The risk of permanent impact on sexual function is a concern for a large proportion (72%) of men (Bouhadana et al., 2020), although this depends on symptom severity, as men with moderate LUTS are less concerned about this than men with mild LUTS (Ikenwilo et al., 2018; Mankowski et al., 2016; Watson et al., 2004).

For many, the decision is not about surgical versus non-surgical options, but rather about choosing between the variety of different surgical options, where men must balance differing possibilities in improving symptoms against differing side effect risks (Gacci et al., 2011; Oelke et al., 2013). For example, while five-year follow-up data for TURP suggest maintained improvements in LUTS and QoL, retrograde ejaculation can occur in around 65% of men treated with TURP (Oelke et al., 2013), and there is growing evidence that impacts on sexual functioning are greater with TURP than with MISTs (Gacci et al., 2011; Lebdai et al., 2019). However, while MISTs appear to provide reasonable improvements in LUTS, there is currently a lack of long-term follow-up data, meaning the durability of some of these treatment options is unclear (Lebdai et al., 2019; Oelke et al., 2013). There are also differences in peri- and post-operative care between different treatment options. Transurethral resection of the prostate requires longer hospital stays than most other surgical options, whilst some MISTs do not require any overnight stay (Oelke et al., 2013). The need for anaesthesia and the length of catheterisation post-intervention also varies between TURP and MISTs, as does the length of time before men start to experience improvements post-intervention (Oelke et al., 2013).

Policy guidance for the treatment of BPH recommends a patient-centred and shared decision-making (SDM) approach, aimed at discussing the risks and benefits of treatment options and patients’ own values and beliefs about diagnosis and treatment (Aubree Shay & Lafata., 2015; Stacey et al., 2017). However, there are various factors that influence whether SDM is practiced with indications that many healthcare professionals do not adopt SDM within routine care (Menear et al., 2018). Further, we have little understanding of how men with BPH balance treatment risks and benefits, or what other factors, not related to side effects or post-operative care, influence men’s treatment choices. Evidence suggests men benefit from support to choose between the different options open to them (Lamers et al., 2020). With indications that the use of tools, such as decision-aids, may reduce men’s decisional conflict or regret, and support a more active role in decision-making, with men’s decisions being more ‘value-congruent’ (van der Wijden et al., 2019).

### The Current Study

Given the various challenges men face in making treatment decisions about BPH, this study aimed to qualitatively investigate the experiences that men have with BPH, and how these relate to treatment choices for BPH. Although there has been some limited qualitative work on QoL issues within this patient group (Ikenwilo et al., 2018; Pateman & Johnson, 2000), there have, to date, only been two other studies which have qualitatively explored men’s decision-making for BPH treatment. One study focussed on men’s concerns around sexual dysfunction in response to watching a video about treatment options for BPH and their side-effects on sexual function (Kelly-Blake et al., 2006). The risk of treatment-induced sexual dysfunction was a major concern for some men, and indicative of resistance to any treatment option, but given the nature of the material used this is perhaps unsurprising; it is also important to note that included participants were experiencing mild/moderate ‘symptom bother’. The second study (Selman et al., 2021) found that clinical assessments such as urodynamics, the experiences of other men patients knew, and perceptions of current symptoms versus potential side effect risks influenced men’s decision-making (Selman et al., 2021). However, the research focused on the role of SDM in BPH treatment and did not present an in-depth analysis of the impact of BPH on QoL, or the barriers and facilitators that men face when choosing whether to proceed to treatment (or not).

Our study therefore extends existing research by presenting a rich and in-depth qualitative exploration of men’s experiences of living with BPH, and their experiences of seeking a consultation on potential treatments for BPH. The study also explores how therapy-related factors (e.g. longevity, side effects) and patient-related factors (e.g. values and experiences around sexual activity, and beliefs and prior experiences of treatments) can influence men’s choice of BPH treatment.

## Methods

### Participants

Participants (*n* = 20) were recruited from urology outpatient clinics at an NHS (i.e. state-funded) hospital in Southern England. Inclusion criteria were that men were aged 45–75 years old, had a diagnosis of BPH (and no comorbid diagnosis of a clinically significant prostate cancer), and had undergone a consultation regarding treatment options within the previous sixmonths. Men were not eligible if they had a diagnosis of dementia or were otherwise impaired in their mental capacity.

Study participants were aged 52–75 years, most (*n* = 19) described their ethnicity as White with one describing their ethnicity as Asian. Participants worked, or previously worked, across a range of sectors, including building, driving, engineering, IT services and the public sector. All participants were in a relationship and most had children (*n* = 17). Additional participant characteristics are provided in Table 1.

**Table 1.** Participant Demographics.

Participant *	Age	Highest educational qualification **	Employment status	Legal marital status	Have any children?
Greg	60	UG Degree	Retired	Married	Yes
Derek	75	GCSE/O-Level/CSE	Retired	Married	No
Josh	52	GCSE/O-Level/CSE	Working	Married	Yes
Paul	57	PG degree/diploma	Working	Married	Yes
Nathan	61	PG degree/diploma	Working	Married	Yes
Scott	66	Diploma	Working	Married	No
Adam	58	A-Level/AS-Level	Retired	Married	Yes
Phil	69	GCSE/O-Level/CSE	Retired	Married	Yes
Matteo	63	UG Degree	Working	Married	Yes
Owen	71	UG Degree	Retired	Single	No
Ryan	70	GCSE/O-Level/CSE	Retired	Married	Yes
Francis	71	GCSE/O-Level/CSE	Retired	Married	Yes
Charles	70	A-Level/AS-Level	Retired	Married	Yes
Ben	74	GCSE/O-Level/CSE	Retired	Divorced/Separated	Yes
Darren	62	Diploma	Working	Widowed	Yes
Ken	70	Diploma	Retired	Single	Yes
Alistair	75	Diploma	Retired	Married	Yes
Jack	71	A-Level/AS-Level	Retired	Married	Yes
Eddie	74	Diploma	Retired	Married	Yes
Ian	72	PG degree/diploma	Retired	Married	Yes

*Note.* *All names are pseudonyms. **PG = postgraduate; UG = undergraduate.

### Procedure

Recruitment involved an initial verbal introduction to the study by clinicians during routine outpatient appointments for their BPH prior to an email referral to the researcher (SG), who provided full study information and arranged interview times. Informed consent was taken either verbally or in writing before interviews commenced. Data were collected using semi-structured, in-depth interviews. Twenty interviews were conducted by SG from September 2020 to August 2021, via video call (e.g. Skype, Zoom, *n* = 7) or telephone (*n* = 13). Each participant was interviewed once, and interviews lasted from 28 to 75 min. Although there was variation in interview length, this is typical of semi-structured approaches and through the analysis, it was apparent that data generated from each interview were rich and meaningful to the overall analysis. SG was the sole interviewer to aid consistency in the process, and experience of, data collection. SG had no prior connection with participants and was not a member of the clinical team. 29 men agreed to be referred, and of these, 20 men participated in an interview. Of the nine men who did not take part, two stated that they had changed their mind about participation, and seven did not respond after receiving the full study information. Recruitment continued until data saturation was reached (Fusch & Ness, 2015; Hennink & Kaiser, 2021).

An interview guide was developed through conversation with urology clinicians, and with reference to BPH literature. Topic areas covered included symptoms experienced, understandings of treatment options and experiences of urology consultations. Example questions included: ‘Tell me about what has led you to this point (of needing to see a consultant)?’ and ‘How do you feel your urinary problems affected you?’. The full interview guide is reproduced at https://osf.io/dqvs3/. An online form (hosted in Qualtrics) was used to collect demographic information from participants. These questions were optional.

The interviewer maintained a sensitivity to participants’ emotions and comfort throughout interviews. To avoid participants feeling ‘forced’ to disclose any information they were uncomfortable sharing, a deliberate decision was taken by the researchers to avoid asking direct questions about whether participants experienced any symptoms related to sexual function. Instead, the interviewer used gentle prompting to further probe during the discussion around symptoms, such as ‘Do you experience any other symptoms, as well as [symptoms already disclosed]?’ or ‘Do you feel the condition has any impact upon those around you, for example, your relationship with your partner or other family members?’. Thereby, any discussion of sexual function arose naturally and was participant-led.

### Data Analysis

Interviews were audio-recorded, and transcriptions were made using an orthographic approach; additional field notes were not taken. Data were analysed inductively using the six-phase process of thematic analysis (Braun & Clarke, 2006). This process involves familiarisation with the data, generating and refining codes, generating initial themes and then developing the final set of coherent yet conceptually distinct themes through discussion and repeated reference to the dataset. A critical realist stance was adopted for data analysis, as this is an epistemological position that reflects the tension between realism and relativism (Potter & Lopez, 2008; Willig, 2008). Critical realism asserts that knowledge and understanding of ‘reality’ can be ‘discovered’ through scientific methods, but that data collected do not themselves provide direct access to such a reality, and the process of discovery and accumulation of knowledge is inevitably socially constructed (Potter & Lopez, 2008; Willig, 2008). As an approach to data analysis, critical realism enables researchers to interrogate and integrate both the subjective realities of participants’ lived experiences as well as the objective realities that can shape and constrain their experiences. Thematic analysis is not rooted in any theoretical, epistemological or ontological position (Braun & Clarke, 2006), and can therefore be applied within a critical realist approach.

Coding and theme generation was conducted in NVivo for Mac (version 12). First, three interviews were independently coded by SG and MH, who then discussed and refined this initial set of codes. SG then coded the remaining dataset and developed a preliminary set of themes. All transcripts were read by MH and DG and the analysis discussed between SG, MH and DG; this was an iterative and interpretative process involving regular discussion and review of coding and thematic concepts to achieve the final analysis.

### Ethics

The study was reviewed by the Cambridge East Research Ethics Committee of the NHS Health Research Authority and was granted a favourable ethical opinion (reference: 20/EE/0095). Research was conducted in accordance with British Psychological Society ethical guidelines and the World Medical Association Declaration of Helsinki, and is reported in line with the Standards for Reporting Qualitative Research (SRQR) (O’Brien et al., 2014).

### Reflexive Comment

It is important to consider the researcher role within research and we acknowledge that data collected are a product of the interaction between interviewer and participant. The sensitive nature of the topic was at the forefront during study planning and implementation. Researcher characteristics (e.g. age, gender, disciplinary background) were discussed with clinicians (Urology Consultants and Nurse Practitioners), with a particular focus on whether the researchers being women would be a barrier to participation and data generation. Clinicians advised that patients encounter clinicians of all genders when seeking treatment for BPH, so would likely be used to talking about their symptoms and concerns regardless of the interviewer’s gender. This appears to have translated into practice with data collection, as evidenced by the rich narratives and broad content covered within the interviews; participants generally spoke candidly about their symptoms, concerns and experiences of treatment. However, we acknowledge the possibility that some individuals may have chosen not to participate, or not to raise certain perspectives, due to the interviewer’s gender.

Drawing on Yardley’s (2000) model for characteristics of ‘good’ qualitative research, a broader consideration to note regards the researchers’ approach to ensuring rigour and transparency in terms of their role within the study. The positioning and preconceptions of all parties were given due consideration with regards their prior experience and understanding of BPH and its treatments. For example, RH as a consultant urologist had no engagement with data generation or analysis, to ensure separation of patient perspectives from the consultant perspective. To enable participants to feel freer in disclosing both positive and negative experiences, the separation between researchers and clinicians was stressed; SG ensured participants understood she was not part of the clinical team and would preserve confidentiality accordingly. SG also advised participants that she was not a medic (but a psychologist) by training, so did not have any clinical perspective on ‘the best’ treatment options. Throughout data collection and analysis, we (SG, MH and DG) discussed our interpretations for consistency, coherence and to ensure the analysis remained grounded within the data; RH’s perspective on the themes was only sought at a late stage once the broad thematic structure and content had been developed. This reflexive approach enabled us to, as far as practicable, bracket off any preconceptions or subjective biases.

## Results

Our participants talked about a range of concerns and issues that were part of the lived experience of BPH, and how these were related to their pathways towards treatment. Here, we focus on four themes that were strongly evident in our data and provide insight into men’s experience and perspectives: ‘Impacts are about more than just physical symptoms’, ‘The path towards treatment’, ‘The process of information gathering’ and ‘Navigating hopes, fears and uncertainty’. Illustrative quotes are provided, with additional data extracts available via https://osf.io/dqvs3/); all names used are pseudonyms.

### Theme 1: Impacts are About More than Just Physical Symptoms

The first theme highlights how the impacts of BPH go beyond the perhaps simplistic framing of BPH as a urinary and sexual function problem, which can be the primary focus for clinicians, to where its effects are felt across a range of social, psychological and practical contexts. Our analyses highlight that, in addition to any direct suffering from physical symptoms, there are broader consequences of living with BPH, which can be far more distressing. For example, many men spoke of significant cognitive burden associated with the physical symptoms of BPH, especially LUTS, in relation to planning where to go (and for how long), of monitoring and adjusting fluid intake, and of changing, adjusting and restricting daily activities:

“It's really meaning that you have to plan, rather than just living… That's how it's impacting on me.” (Nathan).

“We were going to go out on a boat trip…you're out for two or 3 hours…the only way I could even consider that was not to have anything to drink” (Ian)

It’s planning things…I make plans to make sure. I mean, I even put a container in the car in case I get caught short” (Ken).

The anxiety associated with LUTS could be problematic for some men, often centred around access (or not) to toilets: *‘Sometimes I'm about a few miles from home, and because the anxiety is there, I think it makes it worse and then I panic even more and then sometimes I do leak, erm, before I make the toilet.’ (Ken).* This was often exacerbated during the Covid pandemic, where access to public toilets was more restricted, although, for some, the ability to work from home helped address some of the challenges of living with BPH whilst still actively working.

Even where the impacts of BPH physical symptoms are acknowledged by clinicians, these impacts should not be minimised, and more awareness about how physical symptoms are interrelated with cognitive, emotional, social and relational burdens is needed within the clinical consultation. For example, when considering nocturia, some men expressed being unwilling to visit relatives or stay away from home, and they reported negative emotional and social impacts as a result. All men talked about how sleep disruption was an incredibly burdensome aspect of BPH, with some claiming poor sleep as the most detrimental impact on day-to-day QoL: ‘*Very very slowly, the problem gets worse…the biggest issue now is the fact of repeatedly getting up at night, which is a real pain.’ (Derek).* Similarly, what would perhaps be viewed in simple clinical terms as ED had caused significant relationship challenges for some men in our study. Concerns over infidelity and the risk of relationship breakdown within this group was clear:

“The erection thing’s got kind of worse…we went through the stage where my wife thought it was her…she thought I didn't find her attractive anymore, she also thought I was having an affair, which I wasn't…I had to get over that hurdle…then you start worrying…is she going to think, well I'm not getting satisfied at home, maybe I’ll have an affair.” (Josh).

Some participants were candid that living with BPH can take an emotional toll. Embarrassment and shame were common features of men’s emotional experience: ‘*horrible because, you know, [laughs] you've got a big dark patch on your trousers and that’s horrible and you have to live with it’ (Matteo).* Moreover, for some men, living with BPH led to a deterioration in self-esteem and self-worth: *‘I need to get this sorted out because it's, it’s beginning to seriously affect erm, you know, both my self-belief, my self-worth and, and frankly my relationship with my wife as well’. (Paul).* Taken together, these different impacts (physical, cognitive, emotional, social and relational), resulted in significant challenges for men and had affected their QoL across these different domains.

### Theme 2: The Path Towards Treatment

Theme 2 highlights that the process of reaching the point of seeking treatment is quite complex, and often lengthy, with men seemingly moving through three stages of coping: rationalising and minimising the nature of the condition; adjustment and tolerating impacts; until the final stage, where the impact on QoL becomes intolerable (or men fear the condition will progress to becoming intolerable), and men appear open to exploring treatment options.

During the initial phase of symptom onset, people feel there is little to worry about. Symptoms are initially perceived as a relatively minor irritant and not something that causes men much concern or inconvenience. At this stage, men appear to cope partly by rationalising and accepting their LUTS and/or ED as an inevitable part of the ageing process, often with minimal consideration that there is an underlying pathology (prostate-related or otherwise) that could potentially be treated:

“As we get older, things start breaking down and you can't do the things you used to do 20, 30 years ago…then suddenly, you know, after all these years, God, got an aching back, and I’ve this, that, and the other…you know, it's just a sign of ageing.” (Francis)

Two factors seem particularly important in this minimisation. The first is a general relief amongst those with BPH that their condition is not cancerous. There was a sense that once concerns about prostate cancer were ruled out men were more willing to tolerate their BPH symptoms and were less keen to consider invasive treatments. The other factor that seems to contribute to this minimisation is a broader sense of social comparison; participants seemed to feel their position is not as bad as it could be, compared to other people with more severe BPH symptoms or other health conditions:

“I don't think it’s probably as bad as what other people have actually had…my father had something similar…I remember it got to the point where he had to have a catheter put in to deal with it…what I'm going through is nothing compared to what he had at all.” (Scott)

As BPH symptoms start to increasingly impact on their QoL (as discussed in Theme 1) men cope by adjusting across all areas of their life. This adjustment and tolerance of BPH impacts may be partially explained by the perceived reluctance by men in general to seek healthcare advice and treatment (Galdas et al., 2005; Yousaf et al., 2015): *‘It’s that magic combination of men’s health and their reluctance to kind of speak about it and then the fact it's related to you know, manhood and private parts.’ (Adam).* However, there was also, a sense that participants felt by adjusting their behaviours and lifestyle they could effectively tolerate and cope with the symptoms, and indeed most (but not all) within our sample had lived with symptoms for many years. Participants recounted examples of adjusting fluid intake, altering how and when they travelled, or even ceasing engagement in certain activities altogether. However, while these adjustments may seem reasonable and acceptable to participants, that perception is hard to reconcile at times considering the extent of adjustments that men sometimes described:

“I've got to know…the places where I can erm, pull in and go into the bushes…you're organised for things, such as, wearing the right kind of shoes in case when you go into the bushes it's all muddy…or in my little rucksack that I always carry…I always have a repeat prescription…for my medication in case erm, you know, I get stopped by the police or whatever…” (Matteo)

There is a point where men shift to becoming more open to considering invasive treatment options. For most this was a gradual progression, across several years or more, whereas for some men a crisis event, such as a severe urinary infection or relatively severe onset of symptoms, drove treatment seeking more quickly. For all men, however, the data indicates a tipping point is reached, where symptoms are perceived as increasingly impactful and no longer experienced as tolerable: *‘Well, I was thinking this has all gone on long enough... I can’t imagine living another 22* *years or so with this…problem, and one that’s getting progressively worse’ (Paul).* What seemed to drive men reaching this tipping point, was a shift in their perception of symptom severity (and the associated impacts of those symptoms), rather than the length of time that they had lived with and tolerated symptoms. As the condition progressed (whether gradually or more suddenly), men in our sample reappraised their experiences and determined that symptoms and the associated impacts on QoL were becoming less and less tolerable.

### Theme 3: The Process of Information Gathering

As men reappraise their symptoms, and the impacts of those symptoms, as less and less tolerable, they begin to gather information about potential treatment options. Although the procedural aspects of treatments are relevant, the analysis indicated that the focus for men was directed towards the complexities of deciding between treatment options. Given that there are a range of different pharmaceutical and surgical options, and choices vary by location, this can be a confusing process for people.

A key source of information was GPs and urology clinicians. The experience of participants is not always positive, however, with examples of minimisation by health professionals and indications that some GPs represent BPH symptoms as an expected consequence of ageing. Inconsistencies in patient experience across health sites was also evident, for example, with information provision not always clear or consistent between different urology clinics:

“It’s not something er, that is general amongst medical people as to whether you get the correct information or not. I soon found that out. So, for me I found out that the urology nurse talks a lot of sense…[Clinic A] was an experience where the doctors didn’t seem to me to be experienced enough…in urology, compared to [Clinic B], where I ended up. It was 100% different.” (Jack)

But healthcare professionals are not the only source people use to understand their treatment options. Some of this information gathering may involve accessing information online or talking to friends or family members who have had treatment for either BPH or prostate cancer, to understand other people’s experiences of treatment outcomes:

“I saw an article in the newspaper about the steam treatment and I thought, oh, that might be a less invasive way of dealing with things because my main concern was that, if I had erm, any surgery, erm, there would be the, a problem with incontinence.” (Owen).

“My other friend who has just had the Green Laser, we were, you know, just lightly comparing notes.” (Greg).

Participants made it clear that they wanted to have a sufficient understanding of the treatments open to them, so that they felt like they were making an informed decision – but exactly what ‘sufficient understanding’ means varied among participants. For example, some participants were happy to decide based on a single conversation and short explanation of the ‘best’ option for them from the urologist. In contrast, others wanted detailed information on several options, and they also wanted time to go away, reflect, and make further enquiries of their own (e.g. from friends, the internet, an intimate partner) before making their final choice. Such individual differences in terms of ‘need’ during the information gathering process needs to be more fully recognised and supported by all clinicians throughout the treatment journey.

Where confusion arises during this information gathering, people’s confidence in their knowledge and understanding of treatment options may be threatened and they may feel more reluctant to make a choice. Several of our participants spontaneously offered solutions for helping to reduce some of this potential for confusion and improve information provision. These suggestions included additional access to urology specialists, such as having follow-up contact with their urologist after the consultation if they had further questions. Other suggestions included being able to easily access detailed, impartial information about different treatment options in one place (presented in plain English), and the facility to be able to easily understand what treatments are available on the NHS and in which locations.

### Theme 4: Navigating Hopes, Fears and Uncertainties

The final theme highlights individual factors that underpin men’s decision-making around treatment. Although participants were interested to know details about the procedure, such as the type of anaesthetic, how many days they might need a catheter post-operatively, or length of hospital stay, they generally reported that they would not be choosing their treatment based on such procedure-related factors. Instead, there were a range of non-clinical factors that are key to understanding men’s treatment decisions, making it vital to consider the idiosyncratic, individual factors relevant to men. In particular, it is important that treatment options are compatible with work or caring responsibilities; for example, Josh was concerned about dizziness and lethargy as a side effect of medication, as he uses dangerous equipment as part of his job (and yet he indicated this was not something that the consultant had considered or asked about). Other participants talked about ensuring medication did not interact with existing medication taken for other conditions, as well as concerns about how treatment may disrupt plans.

In relation to surgical interventions, participants felt they needed to understand specific risks and benefits, in order to manage their hopes and fears and feel confident about making a decision; these can be summarised as needing an understanding of: (1) the relative risks of certain outcomes across different treatments, (i.e. how likely is incontinence, ED, or other sexual dysfunction for each option), (2) the likelihood of potential improvements (i.e. how likely is the treatment to actually work for them; will their symptoms be meaningfully improved, such that they experience an improvement in QoL), (3) the best time to intervene (i.e. can surgery be performed too early or too late), and (4) the potential longevity of any improvements (i.e. how often they might need repeat procedures):

“What I would expect to be told…is the likelihood of success, any potential side-effects, the recovery times, I want to know as much as possible about each option before a choice was made. I’m not just going to launch into it…it’s something that needs consideration.” (Scott)

There is, of course, some inherent uncertainty around these aspects of the treatment decision process and it was clear from our participants that people differ in terms of how much uncertainty they are willing to risk, and in what areas. For example, some will risk ED but not incontinence, whereas the reverse may be true for others; some are prepared to accept uncertainty about longevity of outcomes for newer treatments, whereas others are not.

For our participants, their concerns about making a treatment choice were focused on their hopes for treatment success and their fears about irreversible side effects. Hopes for likely outcomes could be specific, such as reducing night-time disturbance or regaining sexual function, or more general, related to an overall improvement in QoL. Fears ranged from seeing no improvement in symptoms or seeing some improvement but only for a short time, to fears about full incontinence and ED. In essence, choosing a surgical intervention for BPH involves people managing feelings of anticipated regret about potential ‘catastrophic’ outcomes against their hopes for a realistic, but meaningful, improvement in symptoms:

“It can be about the degree of improvement rather than necessarily an absolute improvement…if they say well, we can do the Rezum and we think it’s going to be a 10% improvement, yeah, I can’t even detect 10% [laughs]…if they say it’s going to completely relieve all your symptoms then I’ll just say, right, do it yesterday.” (Greg)

Overall, there was a sense that men would be more likely to make a treatment choice if they perceived there to be an adequate balance between their hopes and their fears, alongside a perception of an acceptably low level of uncertainty around these hopes and fears. But the hopes and fears vary between men, and over time: *‘If my symptoms were worse, then my threshold for risk would be much higher, but because my symptoms are minor, in my mind, then my threshold for risk is different.’ (Adam)*

Navigating treatment uncertainties can be challenging. Participants expressed the importance of being able to trust their clinicians (whether GPs, nurses, or consultants), for them to have confidence in the advice they receive about treatment options. Central to establishing trust appears to lie in a clinician’s ability to ensure that specific experiences and concerns have been acknowledged and understood: *‘He [urologist] was very, very good. I was very heartened, and he seemed very, he seemed interested in me as a patient, which I thought was quite unusual’. (Paul)*. Positive experiences of consultations involved clinicians who were described by participants as being empathic, understanding, patient, caring, friendly, supportive and attentive; these clinicians had the ability to make people feel listened to and empowered in their treatment decision-making. Although different participants expressed different needs in terms of desired level of agency and the balance of power in SDM, it seems that the more successful consultations were those where the clinician was able to match their style of making recommendations and guiding choice (i.e. fully directive and doctor-led, equally shared with patient, fully delegated and patient-led) to the needs of the person in front of them.

Presenting with BPH can be embarrassing for some people, and clinicians in both primary and secondary care need to be sensitive to this. For some men, there may also be the potential for gender effects (i.e. preferences about talking to a male or female clinician), although participants also expressed how initial embarrassment and awkwardness can be overcome by skilled clinicians. Nonetheless, embarrassment and other negative experiences may be a factor in delaying (or even withdrawing from) help-seeking for BPH. Trust and rapport are fragile and can be easily broken. Dismissive or unprofessional behaviour can be especially damaging and can undermine people’s confidence and increase reluctance in decision-making. For example, some participants felt that clinicians had simply been dismissive of their symptoms, or had not provided the opportunity to discuss the full range of their treatment options and concerns:

“This other bloke [urologist]…it was an awful, it was a bad experience…I just found he was really snooty, he wasn’t listening and…the second I mentioned Rezum, he said, well I don’t do Rezum, that's not me…he wanted to put me on this series of medication and…I said I’m sorry, I’ve tried all manner of medications and he said, well, you know, basically he sent me back home…that was not good.” (Matteo)

In summary, the need for attention to the individual drivers underpinning men’s decision-making is key. Clinicians need to recognise the uncertainties men have and how their hopes for treatment outcomes, or fears about consequences of treatments are idiosyncratic and may change, both between men and within men over time. By helping men navigate the treatment decision-making process it is likely that they will consider different treatments (other than the extremes of nothing versus TURP, for example) but patients’ satisfaction over the consultation and treatment experience will improve.

## Discussion

This study explores men’s experiences of living with BPH, and how therapy-related factors (e.g. side effects) and patient-related factors (e.g. personal values and beliefs about BPH and associated treatments) can influence men’s treatment choices. It provides much-needed insight and patient voice to an under-researched area. Our analyses highlight that BPH can have considerable impact on different aspects of men’s QoL, in ways that go beyond the current framing of BPH as ‘just’ a urination/ED symptom issue. Many men (but not all) tolerate these impacts for several years before presenting to secondary care (up to 20 years within our sample), adjusting their lives to cope with and manage symptoms. The path to the consultant’s door requires men to reach a ‘tipping point’ whereby they feel the current situation is, or is becoming, intolerable. Men’s decisions to proceed with surgery appear to be guided by a balance between their perceptions of their current ability to cope and maintain sufficient QoL, and their individualised perceptions of their hopes and fears for surgery. Being able to trust their clinicians and feel that their concerns have been heard by those clinicians, is also a vital part of men feeling confident in their treatment decisions. To help navigate treatment decision-making, it would be beneficial within consultations to address men’s personal, individualised hopes (of what a successful treatment outcome would be) and fears (of what is the ‘catastrophic’ risk that they perceive).

Our analyses support and extend the findings of a recent systematic review of BPH treatment decisions (Malde et al., 2021). For example, our study similarly highlights that men prefer lower risk treatments with few side-effects, but choices are, at least partially, driven by severity and impact of symptoms. However, this review highlighted that most existing research for treatment decision-making for BPH has focused on influential clinical factors, for example, symptoms of LUTS. Other qualitative or mixed-method studies (Ikenwilo et al., 2018; Kelly-Blake et al., 2006; Pateman & Johnson, 2000; Selman et al., 2021) have indicated that sexual function and impacts on QoL were drivers in treatment decisions, but arguably it was only Selman et al (2021) that attempted to explore broader factors, such as the experiences of other men known to participants. Our research therefore significantly extends the current literature by focussing in-depth on men’s beliefs and experiences up to and including treatment consultation, uniquely providing breadth and depth of insight into both clinical and non-clinical factors that matter to men when making BPH treatment decisions, something which has been called for (Malde et al., 2021).

By using a qualitative approach to foreground men’s voices, our study also contributes to the wider literature by providing additional insight into how the physical symptoms of BPH can have wide-ranging impacts on men’s QoL. As our study was deliberately inductive, we did not adopt a specific a priori definition of QoL; however, our findings suggest that other widely used QoL definitions or frameworks that recognise multi-dimensional aspects of QoL (e.g. SF-36) would be more appropriate for examining the impact of BPH on men’s QoL. Indeed, quantitative studies that have used the SF-36, have found that QoL in men with BPH is lower across all eight domains compared to those in the general population, and sometimes those with other long-term conditions, and that lower QoL is related to greater symptom severity (Hong et al., 2005; Welch et al., 2002)

Our participants reported significant day-to-day challenges that were related to their urinary and/or ED symptoms, and these challenges went beyond ‘just the physical’, affecting their wellbeing and QoL across a range of domains, including the cognitive, emotional, social and relational aspects of their lives. Quality of Life has been defined in different ways within the wider health literature, and the challenges of defining QoL (and health-related QoL specifically) are well-documented (Karimi & John Brazier, 2016). However, within clinical practice consultations for BPH, QoL is typically assessed using a single item measure that is embedded within the International Prostate Symptom Score (IPSS) whereby QoL is directly linked to urinary symptoms. Our findings indicate this is potentially problematic; there is an apparent disconnect between men’s experiences of wide-ranging impacts on QoL and the relatively narrow focus during consultations on physical symptoms, which may reflect the nature of quantitative QoL measurement within health generally. Furthermore, it may mean that factors relevant to treatment decision making and post-operative satisfaction may not be being sufficiently considered.

Our findings highlight several implications for research and clinical practice. Firstly, we have qualitatively documented the range of significant cognitive, emotional and relational impacts on men’s everyday lives, illuminating previous quantitative findings (Girman et al., 1999; Kupelian et al., 2006; Malde et al., 2019). These impacts result in an ever-present burden of planning and worry, varying levels of embarrassment and shame about symptoms, and disruptions to social and leisure activities. However, beyond just documenting these impacts our study additionally highlights how men appear to initially just accept and tolerate their symptoms as an inevitable part of the ageing process and minimise impacts due to their non-cancerous nature. As can be seen in some of our participants, this can result in a significant delay in help-seeking by many men, sometimes for years, with significant further impacts on QoL. This is echoed by other research from the USA that indicates although 90% of men reported LUTS, only 19% sought treatment (Rosen et al., 2003). It is apparent that the pathway from symptom onset via primary care to surgical consultation can be lengthy, at least for some men. There is undoubtedly a need for future research to examine this part of the patient pathway in more detail; both to determine whether length of time from symptom onset predicts treatment choice, but also to better understand whether men need to be further supported within primary care to seek specialist treatment earlier. This might be especially appropriate for those men who have tolerated symptoms for a long time and may have a significantly positive impact on QoL for this group.

Secondly, our study highlights the complex process of treatment decision-making for this group. In terms of choosing to proceed (or not) with a surgical intervention, men appear to be ready to proceed once they have determined that the social, emotional and cognitive burdens have become intolerable, and surgery therefore necessary. Ultimately, however, this depends on each person’s weighing of treatment risks and benefits, and their own assessment of the balance between perceived impacts on QoL and their ability to cope with symptoms. At a basic level, this highlights how and where men need support in making sense of their competing needs and concerns around treatment for BPH. However, there also remain other individual variabilities and uncertainties that warrant further exploration, both in future studies and within the clinical consultation: about surgical risks and associated surgical outcomes (e.g. men’s perceptions about minimally invasive surgical treatments compared to more invasive options), what men hope for in terms of ‘a good improvement’ (this cannot be simplified down to e.g. sexual function and men’s values are idiosyncratic – there are some commonalities but it is certainly not the case that men make choices based on the same priorities), and about the degree of agency in the decision-making that men want.

Finally, there is much scope to work with clinicians to assess the extent to which they are already aware of, and sensitive to, the idiosyncratic needs and concerns of each patient. These needs and concerns are related to questions about treatment options but are also related to those areas of their lives in which men feel BPH symptoms are unacceptably impacting upon their cognitive, emotional, social or relational QoL. Men in our sample felt more confident making the decision to proceed to surgery if they (1) felt they had been given sufficient information about treatment options (including risk of ‘catastrophic’ side effects and likelihood of ‘meaningful’ improvements), and (2) felt supported and ‘heard’ by their clinicians. Trust and rapport between clinician and patient are key to this process. Patient information is therefore relevant, and optimisation of patient information about BPH treatments would be beneficial – but it is important that patient information provision is itself informed by research as an individual’s ability to assess risk accurately depends on how risks are communicated (Garcia-Retamero et al., 2012; Okan et al., 2012; Reyna & Brainerd, 2008).

Each of our participants had different symptoms that they were most concerned by, and each had different hopes and fears for possible ‘best’ and ‘worst’ outcomes from surgery, in terms of physical symptom relief and potential improvements to QoL. These idiosyncratic combinations of current concerns and future hopes and fears need to be considered by those clinicians who are helping patients make their treatment decisions. It is important to recognise possible concerns about the practicalities of delivering an individualised approach within clinical practice – considering the time and financial pressures of health service delivery in non-private healthcare. However, current policy guidance for the treatment of BPH recommends a patient-centred and SDM approach, aimed at discussing the harms and benefits of different treatment options, and integrating patients’ own values and beliefs about diagnosis and treatment (Aubree Shay & Lafata., 2015; Stacey et al., 2017). There are various factors that influence whether SDM is practiced, with indications that many healthcare professionals do not adopt SDM within routine care (Menear et al., 2018) and some evidence of inconsistencies in the extent to which SDM is understood and undertaken across the NHS (Department of Health, 2012). Therefore, further work is needed to ensure an intervention can be developed for use in clinical consultations to enable change in practice to occur.

### Study Limitations

There are limitations in relation to the sample used within this study. All participants interviewed had, to some extent, decided that they would probably proceed with some form of surgical intervention (TURP, MIST etc.), even if the exact choice had not been made. We assume this is reflective of the severity and longevity of BPH for these men, but it is possible that there are men who, with similar clinical symptoms, may defer or decline treatment, and they are not present within this sample. Further, the participants were recruited from specialist urology clinics in Southern England and although men are referred there from across England, we are not suggesting the sample is representative of all men experiencing BPH or seeking treatment for BPH; indeed, there are some individual characteristics, such as different ethnicities, that are not well-represented in our sample. Nonetheless, our sample did include a range of ages, education level and perspectives, providing a depth of insight which we feel does capture well men’s experiences of living with, and seeking treatment for, BPH. Therefore, whilst we were not aiming for our work to be generalisable, it is still likely transferable to other similar clinical contexts with men who are making treatment decisions for BPH. It also helps amplify the patient’s voice within this research domain, thereby informing clinical understanding and attempts to optimise patient-doctor consultations.

## Conclusion

Benign prostatic hyperplasia creates a significant burden and impact on many men’s QoL that goes beyond understanding BPH in purely physical terms of it being a urination or sexual function problem. Seeking treatment for BPH appears to follow a deterioration in physical symptoms and QoL to the point where BPH is no longer tolerable; this is a gradual process for some people, but not for everyone. The decision to proceed with surgical or MIST options appears to be dependent on reaching a tipping point in ability to cope. Findings show that patients are individuals who bring their own set of concerns and preferences to the decision, in terms of which symptoms they are most concerned about addressing, which potential side effects may or may not be tolerable to them and how they assess the risk-benefit profile of the different treatments. Clinicians need to incorporate these individual differences into the SDM for BPH treatment and importantly, clinicians need to not assume what these concerns and preferences are for the individual patient sat in front of them.
